# Reduction in depressive symptoms predicts improvement in eating disorder symptoms in interpersonal psychotherapy: results from a naturalistic study

**DOI:** 10.1186/s40337-020-00308-1

**Published:** 2020-07-03

**Authors:** Malin Bäck, Fredrik Falkenström, Sanna Aila Gustafsson, Gerhard Andersson, Rolf Holmqvist

**Affiliations:** 1 Department of Behavioural Sciences and Learning, Linköping University, Linköping, Sweden; 2 Futurum: Academy for Health and Care, Jönköping, Region Jönköping County Sweden; 3 Faculty of Medicine and Health, University Health Care Research Center, Örebro University, Örebro, Sweden; 4 Department of Clinical Neuroscience, Karolinska Institute, Stockholm, Sweden

**Keywords:** IPT, Interpersonal psychotherapy, Bulimia nervosa, IPT-BN, IPT-BNm

## Abstract

**Background:**

Interpersonal psychotherapy (IPT) can be effective for both Bulimia Nervosa (BN) and co-occurring depression. While changes in symptoms of Eating disorder (ED) and depression have been found to correlate, it is unclear how they interact during treatment and in which order the symptoms decrease.

**Methods:**

Thirty-one patients with BN and depressive symptoms received IPT using the manual IPT-BNm in a naturalistic design. The outcome was measured with the Eating Disorder Examination Questionnaire (EDE-Q) and the Montgomery Åsberg Depression Rating Scale (MADRS-S). Symptom improvement at each session was measured with Repeated Evaluation of Eating Disorder Symptoms (REDS) and the Patient Health Questionnaire-9 (PHQ-9).

**Results:**

Significant improvements with large effect sizes were found on both ED symptoms and depression. The rates of change were linear for both BN and depression. A strong correlation between reduction of depressive symptoms and ED symptoms was found. Depressive symptom reduction at one session predicted improvement of ED symptoms at the next session.

**Conclusions:**

IPT-BNm had an effect on both BN and co-occurring depressive symptoms. The analyses indicated that reduction in depressive symptoms preceded reduction in bulimic symptoms.

## Plain English summary

Interpersonal Psychotherapy (IPT) is an effective treatment for major depression and eating disorders. In this study, 31 patients suffering from Bulimia nervosa (BN) and co-occurring depression received 16 weeks of IPT. In the therapy, bulimic symptoms were linked to on-going interpersonal events. The therapy is based on the assumption that if patients’ interpersonal stress is reduced, if they receive social support, process their feelings, and improve interpersonal skills; their eating disorder symptoms will decrease. The results in our study showed that the treatment was effective for symptoms of BN and depression. The average rate of change was linear. Even though early response was associated with favourable treatment outcome, the effect took place continuously during the whole treatment, indicating that there were different paths to recovery. Symptom improvement in BN and depression was strongly correlated. Reductions of depressive symptoms at one session were correlated with a reduction in eating disorder symptoms at the next session. As comorbid depression is common in eating disorders (ED) and may jeopardize the ED treatment, this is an urgent topic and further research is needed to validate the findings of this study.

## Background

Patients with Bulimia Nervosa (BN) often have recurrent relapses and a life-long vulnerability for eating disorder (ED) behaviour. Even with recommended treatments, only about half of the patients recover [1, 2]. One factor that may complicate treatment of BN and has a negative effect on the outcome is the high co-morbidity with depression [3]. The association between depression and ED symptoms can be intricate, with depression initiating eating problems for some patients and eating problems initiating or worsening a depression for others. Often, the relationship is best described as a vicious circle between these symptoms. A meta-analysis on the longitudal relationship between ED-pathology and depression showed a bi-directional relationship, indicating that depression and ED affect each other, and that they might be influenced by shared risk factors [4]. In the light of these findings the next important step is to understand how the two conditions influence each other during ED-treatment. Cognitive Behavioural Therapy (CBT) is the primary recommended psychological treatment for patients with BN and binge eating disorder (BED) [5]. In the most studied form of CBT treatment for bulimia [6], it is recommended that co-occurring depression should be treated before focusing on ED [6, 7], given that mood intolerance as well as interpersonal difficulties, low self-esteem and perfectionism may be maintaining processes that inhibit ED treatment [6]. Interpersonal psychotherapy (IPT) has been suggested as a valuable treatment option for patients with BN or BED, as IPT focuses particularly on such problems as mood and interpersonal stress [6, 8, 9].

In a previous study using an IPT manual for depression, the authors examined the effect of IPT on ED with co-morbid major depression and found significant improvements with large effect sizes on both ED and depression measures [10]. Symptom reductions for the two diagnoses were strongly correlated, which indicate that the mechanisms of change in IPT for depressive symptoms may also work on ED symptoms or that change in one syndrome promotes change in the other. There is limited research on whether improvement in depression during psychological treatment precedes improvement in ED symptoms, or if it is the other way around with improvement in ED symptom preceding improvement in depression.

IPT was created as a treatment for depression and has also shown positive results for other mental disorders, such as social anxiety disorder, post-traumatic stress disorder (PTSD) and ED [11–13]. In ED research, IPT was first used as a control treatment for CBT in a randomised controlled trial on individuals with BN [14]. In this trial, Fairburn et al. (1991) used an IPT-manual that was not specifically adapted for BN. Beyond initial psychoeducation; eating problems were not addressed during the treatment. However, as the IPT treatment showed positive results, a manual called IPT-BN was created [15]. Using this manual, positive results have been attained in both individual and group settings for patients with full or sub-threshold BN and BED [7]. In comparison with CBT, IPT seems to have a delayed effect on ED-symptoms. Long-term follow-up studies have shown that patients who have been treated with IPT continue to improve after treatment [8, 9, 16]. IPT focuses on current interpersonal stressors associated with ED symptoms [7, 17]. The interventions in IPT promote interpersonal problem solving, emotional processing and practicing communication of need of social support [7, 18], which can help patients improve their interpersonal life and may have a positive effect on their self-image [7]. This is supposed to lead to improvements in ED-symptoms secondary to relational improvements. The fact that IPT only indirectly focuses on behavioural change may lead to slower changes in eating behaviours [19].

A central component in IPT is the explicit exploratory linking between current symptoms and relational issues [20, 21]. However, the original IPT-BN manual did not include this central aspect of IPT [7, 9]. There was also no monitoring of ED symptoms after the initial sessions, as is usually the case in IPT (see below and under Method section). Techniques such as problem solving were not used. This has been proposed as another explanation for the delayed treatment response in IPT-BN compared to CBT [22]. In order to improve IPT for BN, a manual was created where the ED problems were in focus by including original components of IPT such as psychoeducation, weekly evaluation of current symptoms (in this case eating problems), directive techniques, problem solving, modelling and role play [23]. In this modified version of IPT-BN (IPT-BNm; Wight et al., 2011 [23], ED symptoms are viewed as interpersonal markers that are monitored and discussed during each session. For instance; if the therapist and patient note that the patient has deviated from the intended schedule of food and exercise, or if the patient reports changes in frequency of binge eating or vomiting, the therapist encourages exploration of potentially emotionally stressful interpersonal events that may have preceded change in ED symptoms. In a deepened cyclic process, explicit conceptualisations of associations between ED symptoms and interpersonal events are being made. The aim is to facilitate awareness, hope and resolutions in the chosen interpersonal focus area [18].

Two studies have shown initial support for this treatment [24, 25]. An open trial by Arcelus including 59 patients with BN found significant reductions in ED symptoms at the end of treatment [25]. The three-month follow-up found that the symptom reduction had continued. The patients also suffered from co-morbid moderate depression, which improved significantly during treatment [25].

In many psychotherapies, rapid symptom reduction predicts final treatment response [26]. The preliminary results of IPT-BNm showed a more rapid response [25] compared to earlier studies of IPT-BN by Agras [8]. By the middle of therapy (session 8), the patients had made significant improvements in terms of ED and depressive symptoms [25]. Furthermore Arcelus et al. compared a brief form of IPT-BNm with 10 sessions (IPT-BN10) with IPT-BNm (16 sessions) and a waiting-list control group in a small-randomised trial including 30 patients. Significant differences were found for patients treated with IPT-BN10 and the waiting-list control. No significant differences were found on any of the EDE-Q scales when comparing IPT-BN10 and IPT-BNm. The authors suggested that IPT-BN10 may work faster than previous research on IPT-BN has shown and that IPT also can be delivered in a briefer format [24].

The primary aim of this study was to evaluate the effectiveness of IPT-BNm by Whight et. al [23]. on ED symptoms and depressive symptoms in a Swedish treatment context. In addition, we also analysed a) whether early improvement in ED symptoms and/or depressive symptoms predicted better outcome, and b) whether change in ED symptoms preceded change in depressive symptoms or vice versa.

## Methods

Thirty-one bulimic patients were included in this naturalistic study, which was performed in conjunction with an implementation project by the Swedish Eating Disorder Register (SwEat) in IPT-BNm. The data collection was conducted at seven outpatient psychiatric services specialising in ED treatment in Sweden. The patients received ED focused IPT with an explicit focus on the links between interpersonal issues and ED symptoms, according to the manual IPT-BNm (23, 24, 25,). Therapy sessions were videotaped, and the supervisor rated at least three sessions in every therapy for competence and adherence. According to the adherence rating procedure (supervisor ratings), the therapists conducted IPT-BNm in an adherent and competent way [20]. The Ethics Review Board in Örebro/Uppsala approved the study 2012-12-05, registration number; 2012/471. Written informed consent was obtained from all participants.

### Participants

#### Patients

Inclusion criteria were age 16–50 years and meeting the DSM-IV criteria for Bulimia nervosa [27]. The frequency and duration criteria were not used. Exclusion criteria were a body mass index (BMI) lower than 17.5, other on-going psychological treatment and/or inpatient care, a need for intense medical treatment or drug abuse. Thirty-one bulimic patients with co- occurring depressive symptoms were included in the study. The patients were recruited at outpatient services where the therapists worked. All 31 patients completed the treatment. They were all women, in the age between 19 and 50 years. Patients were initially assessed at the ED services according to the routine by SwEat (SEDI and EDE) [28], which was complemented with a clinical interview made by the therapist in a pre-treatment assessment session. This assessment session was part of the treatment protocol of the study, called *session zero* and it included, among other items, questions based on the DSM-IV [27], the self-assessment instruments EDE-Q and REDS. Even if co-morbid depression was not an inclusion criterion, the patients were also assessed for co-occurring depressive symptoms based on the MADRS-S and PHQ-9. The patients were diagnosed with Bulimia nervosa [27]. They also presented a mild or moderate depressive psychopathology. For most of the patients, this was their first BN treatment. However, according to the medical records, five of the patients had had a former assessment more than 6 months earlier, so they may have received treatment-related information/psychoeducation earlier.

#### Therapists

Seventeen therapists participated in the study. Nine therapists had one patient and eight therapists had two to four patients. The data collection was conducted during the years 2013–2016, and was made in conjunction with an IPT-project by SwEat which had the aim to teach therapists IPT-BNm [22, 23]. The therapists received weekly internet-based group supervision by accredited IPT-supervisors. All 17 therapists were experienced ED therapists before starting the IPT training but had not conducted IPT therapies focusing on ED symptoms using IPT-BNm. All had completed at least one previous IPT-therapy with a depression focus for a patient with co-occurring ED [20, 21]. One third of the therapists had previous training in CBT, one third had training in psychodynamic therapy and one third had an integrative training. Ten therapists were clinical psychologists, three were social workers, three were occupational therapists, two were nurses, and one auxiliary psychiatric nurse. Therapists were between 31 and 61 years old and had between 2- and 16-years’ experience of psychotherapeutic work. Among those therapists who collected data in more than one IPT-therapy, they all had the opportunity to receive regular supervision and had support from their managers and workplaces to work with IPT and make preparations for data collection and supervision.

### Description of the treatment IPT-BNm

Interpersonal psychotherapy for Bulimia nervosa modified version, IPT-BNm, consists of 12–16 weekly sessions of 45 min duration. The initial four sessions cover a detailed assessment and review of the patient’s ED symptomatology, linking to the interpersonal context and how that maintains the ED. The initial phase also provides psychoeducation regarding patients’ ED and associated symptomatology. A timeline of symptoms and life events is also completed along with an interpersonal inventory. In collaboration between patient and therapist, an interpersonal focus area is chosen that will form the basis of the work of the treatment phase. IPT recognizes four focus areas: interpersonal dispute, interpersonal role transition, complicated grief and interpersonal deficit/sensitivity. The treatment phase consists of discussing the relationship between the interpersonal focus area and current ED symptoms. ED symptoms are tracked during each session. Specific techniques used during these sessions include: communication analysis, role-play, clarification, exploration and encouragement of affect, use of the therapeutic relationship and behavioural change techniques; such as decision analysis and problem solving. In the final two sessions explicit discussion of the ending of therapy takes place as well as contingency planning for the future and a reflection on the interpersonal skills learned during therapy [23].

### Outcome measures

*The Eating Disorder Examination Questionnaire* (EDE-Q) is a widely used self-report measure of ED psychopathology [29, 30], derived from the EDE- interview [31], and has satisfactory validity and reliability [32, 33]. In addition to a total score, the EDE-Q generates four subscales; Restraint, Eating Concern, Shape Concern and Weight concern. Clinical norms for a female Swedish bulimic population have suggested cut off as following scores; Restraint: M = 2.9 (SD = 1.1), Eating Concern: M = 2.9 (SD = 0.9), Shape concern: M = 4.4 (SD = 1.3), Weight Concern: M = 3.4 (SD = 1.6), and finally EDE-Q total: M = 3.4 (SD = 1.0) [34]. EDE-Q was administered at the first, eighth and last session.

The *Montgomery Åsberg Depression Rating Scale* (MADRS-S) is the self-rated version of MADRS, especially developed to be sensitive to changes in depression severity [35]. The MADRS-S consists of nine items corresponding to the nine DSM-IV major depression criteria. By MADRS-S standards, 13–19 points indicate mild depression, 20–34 moderate depression, and more than 34 points severe depression [36]. The instrument, which has good reliability and validity [35], was administered at the first, the eighth and the last session.

The *Repeated Evaluation of Eating Disorder Symptoms* (REDS) is a self-rating questionnaire, which measures the most common ED-symptoms [37]. The 14 REDS items comprise thoughts and ideas about food, impulses, weight and body image. The patient gives responses on a 5-point scale ranging from ‘never’ to ‘very often’ and indicates how often he/she has experienced each symptom during the past week. The REDS has demonstrated good reliability and validity in a Swedish study [37]. The cut-off between clinical and non-clinical populations is 22 points. In this study, REDS was used weekly to measure symptom changes. The REDS was completed before each session.

The *Patient Health Questionnaire-9 (*PHQ-9) consists of questions corresponding to the nine DSM-IV major depression criteria [38]. The PHQ-9, a validated and reliable instrument, has proved suitable for screening as well as repeated measurements. The cut-off limits for symptom severity are: 0–4 none/minimal depression, 5–9 mild depression, 10–14 moderate depression, 15–19 intermediate depression and 20–27 severe depression [39–41]. A cut-off score of 10 points has been suggested for differentiating clinical from non-clinical individuals [42, 43]. This study used the PHQ-9 to measure weekly symptom changes and was completed before each session.

### Data analysis

Paired samples *t*-tests were used to compare the start and end values. Effect sizes were calculated as within-group Cohen’s *d*. If patients completed their treatment before the sixteenth session, last observation carried forward (LOCF) was used for the final PHQ-9 and REDS scores. Reliable change was assessed using the Reliable Change Index (RCI) [44]. The RCI indicates whether the symptom change exceeds measurement error. Jacobson and Truax’s third definition was used to determine the RCI with Cronbach’s alpha used as estimation of the reliability [45]. Seven points on the REDS and five points on the PHQ-9 were found to be RCI limits by this method. The cut-off for clinical significance (CS) was 10 on the PHQ-9 and 22 on the REDS. Patients who improved more than the RCI limits and also scored below the CS cut-off after treatment were considered to have attained a reliable, clinically significant change and were considered *remitted* [44], while patients with reliable change who did not reach the CS level were classified as *improved*. Patients whose symptoms increased more than the RCI limit were labelled *deteriorated*, and the remaining patients were labelled *unchanged*. The significance of early response was tested by regression analyses and by testing correlations between early and final responders. Patients who had reached a reliable symptom change at session four (REDS with seven or more point and on PHQ-9 with five or more points) were considered as early responders. Session four was targeted based on that the interpersonal focus area in IPT should be formulated in this session and is the final session in the initial phase [21]. Final responders were also defined of reliable symptom change (RCI). Pearson correlations were used to examine associations between outcome scores for eating disorder (REDS) and depression (PHQ-9). Within-patient analyses were done using Dynamic Structural Equation Modeling (DSEM). DSEM is based on time-series analysis but can be used with panel data due to the possibility of adding random effects to model between-patient components [46]. As such, DSEM is a kind of Multilevel Model (MLM), but with two important differences to traditional multilevel models: 1) random effects can be modeled for both predictors and outcome variables, and 2) lagged effects are modeled using latent variables [47]. These two additions are essential for avoiding the so-called dynamic panel bias, which arises when regressing a variable on its own lagged component, while using manual methods for separating within- and between patient components [48]. The part of the DSEM model of most interest is the within-patient part, which is a cross-lagged panel model in which lagged time-series relationships among variables are estimated. The within-patient cross-lagged paths, which are the ones of most interest in this study, represent the average effect of one variable at any given session on the other variable by the following session. This path is adjusted for autoregression, i.e. the effect of the outcome variable at the prior occasion. This is important since it means that the cross-lagged effects can be interpreted as effects on (residualized) change in the other variable. Specifically, in the present study the relationships between depression and ED symptoms are analyzed. At a between-patient level, different patients may have different average levels of depression and/or ED symptoms across the whole study. It may also be that different patients have different linear trajectories across the whole study, in that some improve more/faster, while others remain more or less on the same level or even deteriorate. At a within-patient level, we analyze whether deviations from each person’s average level (or average linear trajectory over time) in depression/ED at a given session can predict change in depression/ED by the next session. This is a test of which symptom ‘drives’ change in the other symptom. DSEM models are estimated using Markov Chain Monte Carlo estimation, which is a simulation-based estimation method for Bayesian analysis. Two chains were run, and Potential Scale Reduction value (PSR) was calculated to check for convergence between the chains. PSR values were required to be below 1.05 and remain so for at least 2000 iterations. Estimation was done using Mplus version 8.1.7, with default infinite variance priors to ensure that analyses are not unduly influenced by prior information [49]. In panel data, there are two dimension of sample size, the number of participants (N) and the number of repeated measurements (T). For within-person coefficients, statistical power and coefficient bias are determined roughly by the product of these dimensions. A recent simulation study [50] on a similar SEM model as the one used in the present study showed that with *N* = 50 and T = 5, i.e. 250 observations in total, statistical power was well above 80% for finding a medium-sized effect, and average coefficient bias was negligible. In the present study we had *N* = 31 and T = 16, i.e. 496 observations, which should be sufficient even for smaller effect sizes.

## Results

### Eating disorders and depressive symptoms outcome

As shown in Table 1, both ED symptoms and depressive symptoms decreased significantly, with large effect sizes, during the treatment. The patients improved significantly on all EDE-Q subscales from a clinical to a non-clinical level, with large effect sizes.

**Table 1:** Pre- and post-ratings of ED and depressive symptoms for the 31 patients

Measure	Pre- Treatment		Post-Treatment		*t*	*p*	Cohen’s *d*
	M	SD	M	SD			
Eating disorder problems:							
EDE-Q							
Global	3.95	0.76	2.38	1.36	5.32	<.001	1.48
Restraint	3.55	1.48	1.88	1.36	4.70	<.001	1.17
Eat concern	3.7	1.4	1.7	1.26	4.64	<.001	1.5
Shape concern	4.65	1.4	3.28	1.89	4.00	<.001	0.83
Weight concern	3.86	1.24	2.64	1.62	3.30	.003	0.85
REDS	32.58	9.69	15.52	8.24	8.59	< .001	1.90
Depression:							
MADRS-S	18.24	8.91	11.30	5.97	4.15	< .001	0.93
PHQ-9	13.81	5.34	5.48	4.53	7.36	< .001	1.68

*Note: EDE-Q* Eating Disorder Examination Questionnaire, *REDS* Repeated Evaluation of Eating Disorder Symptoms, *MADRS-S* Montgomery Åsberg Depression Rating Scale and PHQ-9 = Patient Health Questionnaire-9

Ratings on the EDE-Q, REDS, MADRS-S and PHQ-9 are presented in Table 1.

### Symptom changes during treatment

**Fig. 1:**
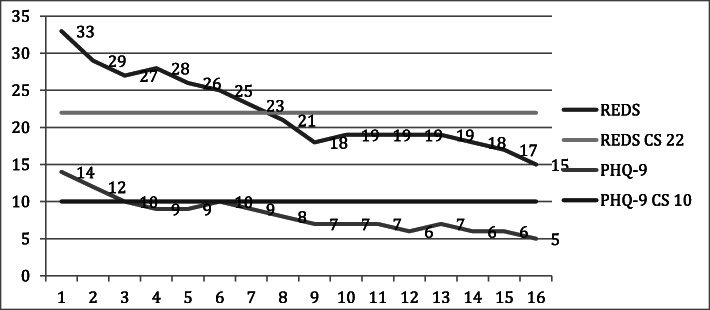
Symptom trajectories for REDS and PHQ-9. REDS = Repeated Evaluation of Eating Disorder Symptoms, PHQ-9 = Patient Health Questionnaire-9, and CS = cut off score for clinical significance

In Fig. 1, symptom improvement on REDS and PHQ-9 are shown.

The symptom reduction of ED (REDS) during treatment was linear, with the standardized beta coefficient − .45 (*t* [28] = − 11.16, *p* < .001). Symptom improvement was marginally larger in the first part of the therapy than in the second, with a reduction of 10 points to session 8 and a further reduction with 8 points to session 16 but a quadratic regression model was not significant. The average participant reached the cut off score of 22 for REDS at session nine. The symptom reduction of depression (PHQ-9) was also linear with the standardized beta coefficient − .41 (*t* (30) = − 9.41, *p* < .001). The quadratic coefficient was not significant.

### Reliable symptom change

The results in Table 2 show the numbers of remitted, improved, unchanged and deteriorated patients.

**Table 2:** Numbers of deteriorated, unchanged, improved and remitted patients (*n* = 31)

	Deteriorated	Unchanged	Improved	Remitted
REDS	1	6	5	17
PHQ-9	1	7	0	23

Note: *REDS* Repeated Evaluation of Eating Disorder Symptoms, and *PHQ-9* Patient Health Questionnaire-9

Twenty-two of the 31 patients were remitted [17] or improved [5] in their ED-symptoms. Two more passed the cut-off for CS [22] but were not considered remitted due to low entry values. Six remained unchanged and one had reliably deteriorated in ED symptoms. However, this patient went from clinically depressed to remitted in depression.

Fifteen patients were remitted [12] or improved [3] in both ED and depression. All but two patients were below the clinical level of depression at termination of treatment. Twenty-three were remitted and another five were below the clinical level but were not considered remitted because of low entry values. Among the two patients who were still clinically depressed, both had achieved a reliable change in ED symptoms.

### The relationship between early response and final outcome

In order to study the significance of early response an assessment of patients’ symptom change at session 4 was made. Those who had reached a reliable change in ED-symptoms at session 4 on REDS (change ≥7 points) were considered as early responders. The association between early responders and final responders was close to significance (χ^2^ (1) = 3.70, *p* = .054). There were seven early response patients; all of those were also final responders. There were 13 early responders on PHQ-9 (change ≥5 points); 12 of them were also final responders. The association between early response and final responding was significant (χ^2^ (1) = 3.84, *p* = .05). Early response on REDS was not associated with final responding on PHQ-9 (*p* = .43); early response on PHQ-9 was not associated with final responding on REDS (*p* = .56).

### Associations between symptom change in ED and depression

Change in gain scores on ED-symptoms (REDS) and depressive symptoms (PHQ-9) were strongly correlated (*r* = .60, *p* = .001). In order to further analyze the associations between ratings on REDS and PHQ-9 between sessions, the associations were analysed using DSEM. Model 1 was a basic cross-lagged panel model, with REDS and PHQ-9 at session *t-1* predicting REDS and PHQ-9 at session *t*. In Model 2, we adjusted for linear change over time in both variables. REDS and PHQ-9 were standardized before analysis, to make coefficients for these comparable. Results are presented in Table 3. The coefficients of most interest in Table 3 are the cross-lagged within-patient effects, i.e. REDS_t-1_ → PHQ-9_t_ and PHQ-9_t-1_ → REDS_t_. As can be seen from Table 3, in Model 1 both of these paths were statistically significant. Although the REDS_t-1_ → PHQ_t_ path seemed numerically larger, the difference between these two coefficients was not itself statistically significant (difference = 0.11, se = 0.08, *p* = 0.18, 95% CI -0.05, 0.26). In Model 2, which adjusts coefficients for the person-specific linear slope of session number, the PHQ_t-1_ → REDS_t_ path remained about the same as in Model 1 (0.13, se = 0.06, *p* = .04 95% CI 0.00, 0.25), while the REDS_t-1_ → PHQ_t_ path disappeared completely (− 0.01, se = 0.08, *p* = .92, 95% CI -0.16, 0.16). Still, the difference between these two coefficients remained statistically non-significant (− 0.14, se = 0.12, *p* = .26, 95% CI -0.35, 0.10).

**Table 3:** Fixed effects from Dynamic Structural Equation Models testing the session-wise lagged relationships between symptoms of eating disorder and depression

	Model 1				Model 2			
Fixed effects, within level	Estimate	sd	*p*	95% CI	Estimate	sd	*p*	95% CI
PHQ_t-1_ → PHQ_t_	0.58	0.05	<.001	0.48, 0.69	0.44	0.08	<.001	0.29, 0.59
REDS_t-1_ → PHQ_t_	0.24	0.05	<.001	0.14, 0.34	−0.01	0.08	.92	−0.16, 0.16
REDS_t-1_ → REDS_t_	0.73	0.05	<.001	0.63, 0.83	0.20	0.08	.02	0.04, 0.36
PHQ_t-1_ → REDS_t_	0.13	0.05	.008	0.04, 0.23	0.13	0.06	.04	0.00, 0.25
PHQ_t_ ↔ REDS_t_	0.15	0.017	<.001	0.12, 0.19	0.12	0.01	<.001	0.10, 0.16
Fixed effects, between level								
PHQ_intercept_ ↔ REDS_intercept_	0.089	0.211	0.52	−0.10, 0.69	0.68	0.41	.01	0.15, 1.71
PHQ_slope_ ↔ REDS_slope_					0.00	0.00	.04	0.00, 0.01
PHQ_intercept_ ↔ REDS_slope_					−0.02	0.02	.26	−0.07, 0.02
PHQ_slope_ ↔ REDS_intercept_					−0.02	0.02	.08	−0.07, 0.00
PHQ_intercept_ ↔ PHQ_slope_					−0.03	0.02	.002	−0.09, − 0.01
REDS_intercept_ ↔ REDS_slope_					−0.06	0.03	<.001	−0.13, − 0.02

Note. PHQ_t-1_ and REDS_t-1_ are the PHQ-9 and REDS scores, respectively, at session t-1, while PHQ_t_ and REDS_t_ are the PHQ-9 and REDS scores at scores at session t. Unidirectional arrows (→) represent regression paths (potentially causal), while bidirectional arrows (↔) represent covariances

## Discussion

The aim of this study was to analyse the effects of IPT on symptom reduction in patients with bulimia nervosa with co-occurring depression. The results indicate that the treatment was effective. Based on EDE-Q results, patients on average improved from a clinical level in ED symptoms to a non-clinical level. This finding is in line with previous research showing that IPT is an effective treatment for BN. Effect sizes were similar to those in previous IPT-studies regarding BN [8, 9, 25]. Both ED symptoms and depressive symptoms decreased substantially and significantly, with most patients reaching clinically significant change. Twenty-four (77%) of the 31 patients were remitted or improved in bulimic symptoms at the end of treatment. Fifteen of the patients (48%) were remitted or improved on both diagnoses.

There was a trend for patients with early response to improve to a larger extent than the other patients. These results are parallel to results of CBT for BN, where early responding also usually predicts final outcome [7, 14, 17]. However, the clinical implications of this finding must be used with caution since the improvement trajectories differ between patients in this study and a number of patients improved although they were not early responders. It has been suggested that IPT could be delivered in a briefer format, since many of the patients in the studies by Arcelus [24, 25] improved more during the first eight sessions. However, ED often implies life-long vulnerability with recurrent relapses [1] and thus it could be argued that IPT-BNm of 16 sessions is not an unnecessary extensive intervention, especially if the treatment targets several co-occurring conditions. In our study, symptom reduction was linear during the whole treatment, and there was no indication that improvement took place at different rates early and late in treatment. Research suggests that patients improve in different rates but on the average linearly [51] and that the long-term effects of IPT are stable [15, 16, 19]. Downsizing treatment duration may therefore not be cost-effective in the long run. Maybe the question we should be asking ourselves is how well the results can be maintained, rather than the speed of recovery.

There is a substantial comorbidity between ED pathology and depression [4]. The relations between these syndromes are intricate, as depression may initiate eating problems and eating problems may initiate a depression. Often, the development is a vicious circle between these symptoms. In CBT treatment of comorbid ED and depression, therapists are often encouraged to first help the patient with the depression, as depressive thoughts and feelings may hamper the therapeutic work with the eating behaviour [6]. In order to enhance effective treatment of combined conditions, it is important to better understand whether decrease in depression predicts improvement of eating problems, or it is the other way, i.e. ED improvement precedes improvement in depressive symptoms. The cross-lagged analyses of symptom reduction showed that when adjustment for individual linear change trajectories was made, reduction in depressive symptoms predicted reduction in ED symptoms at the next session whereas reduction in ED symptoms did not predict reduction in depressive symptoms. The difference between these coefficients was not significant, probably due to low statistical power. It can be argued that depression should be targeted before ED symptoms in comorbid conditions [6]. General models of psychotherapy emphasize the need to restore hope and motivation in the patient before more specific symptoms are focused [52, 53]. In this study, decrease in depressive symptom at one session was correlated with decrease in ED symptoms at the next session. IPT-BNm focuses especially on ED symptoms. The fact that the treatment was also effective for depression, and that reduction in depressive symptoms seemed to correlate with subsequent reduction in ED symptoms, is interesting. One perspective is that IPT, by focusing on interpersonal issues, has a more general impact on emotional suffering than treatments that target specific symptoms. The treatment can have a remoralizing effect, influencing depressive symptoms such as hopelessness, alienation and distrust. If interpersonal issues become more understandable and manageable, a general reduction of the sense of being stuck and hopeless might be achieved. A reasonable suggestion is that the treatment first leads to a decrease of depressive symptoms and subsequently reduces ED-symptoms. Furthermore, since the IPT approach focuses on processing interpersonal stressors and negative affects which is hypothesized to lead to improvements in ED symptoms [7], it seems natural that this process of change may appear in different pace depending on the patients’ interpersonal context. Thus, more research concerning what, how and when change takes place in IPT for eating disorders needs to be done. This is the first study to our knowledge to analyse correlations between weekly ratings of symptoms in co-occurring conditions, both with regard to ED treatment in general and in IPT research. Although this study is small, these results suggest that change in depressive symptoms is strongly correlated with change in ED symptoms the next session. Since depressive co-morbidity with BN is common and may complicate or obstacle ED treatment [4, 6], this is a clinical important finding showing that IPT-BNm may be an especially suitable treatment option for patients suffering from BN and co-occurring depression.

### Strengths and limitations

The main strength of this study was the weekly ratings of symptoms, which made it possible to analyse the interaction in symptom reduction. The study was naturalistic with a limited number of patients. This may obstruct the generalizability of the results. Another limitation was the lack of follow-up data. Since the patients presented with mild to moderate depressive symptomatology, we do not know whether the findings can be generalized to more severe forms of mood disorders. For future studies, it would be important to measure change in identified interpersonal problems.

## Conclusions

The modified version of IPT-BN used in this study is an effective treatment for BN, especially with co-occurring depression. Symptom improvement in ED and depressive symptoms was strongly correlated, with reduction of depressive symptoms at one session driving symptom reduction in ED at the next session. Even though early response was associated with favourable treatment outcome, effect took place continuously during the whole treatment, indicating that there were different paths to recovery.

## Data Availability

The datasets used and/or analysed during the current study are available from the corresponding author on reasonable requests.

## Abbreviations

APA: American psychiatric association
BED: Binge eating disorder
BN: Bulimia Nervosa
BMI: body mass index
CBT: Cognitive Behavioural therapy
CS: Clinical significance
ED: Eating disorder
EDE: Eating Disorder examination
EDE-Q: Eating Disorder examination Querstionnarie
DSEM: Dynamic Structural Equation Modelling
DSM-IV: Diagnostic and statistical manual of mental disorders - 4th edition
IPT: Interpersonal Psychotherapy
IPT-BN: Interpersonal Psychotherapy for Bulimia nervosa
IPT-BNm: Interpersonal Psychotherapy for Bulimia nervosa, modified version
IPT-BN10: Interpersonal Psychotherapy for Bulimia nervosa, ten sessions
LOCF: Last observation carried forward
MADRS-S: Montgomery Åsberg Depression Rating Scale
MLM: Multi level model
PHQ-9: Patient Health Questionnaire-9
PSC: Potential Scale Reduction value
PTSD: Post traumatic stress disorder
RCI: Reliable change index
REDS: Repeated Evaluation of Eating Disorder Symptoms
SEDI: Structured Eating Disorder Interview
SWEAT: Swedish Eating Disorders clinical quality register
